# The Stimulation Effect of Auricular Magnetic Press Pellets on Older Female Adults with Sleep Disturbance Undergoing Polysomnographic Evaluation

**DOI:** 10.1155/2013/530438

**Published:** 2013-03-21

**Authors:** Chyi Lo, Wen-Chun Liao, Jen-Jiuan Liaw, Liang-Wen Hang, Jaung-Geng Lin

**Affiliations:** ^1^School of Nursing, China Medical University, Taichung 40402, Taiwan; ^2^Department of Nursing, China Medical University Hospital, Taichung, Taiwan; ^3^School of Nursing, Chung Shan Medical University, Taichung, Taiwan; ^4^School of Nursing, National Defense Medical Center, Taipei, Taiwan; ^5^Department of Respiratory Therapy, China Medical University, Taichung 40402, Taiwan; ^6^Sleep Medicine Center and Department of Internal Medicine, China Medical University Hospital, Taichung, Taiwan; ^7^School of Chinese Medicine, China Medical University, No. 91 Hsueh-Shih Road, Taichung 40402, Taiwan

## Abstract

*Study Objectives*. To examine the stimulation effect of auricular magnetic press pellet therapy on older female adults with sleep disturbance as determined by polysomnography (PSG). *Design*. Randomized, single-blind, experimental-controlled, parallel-group. *Setting*. Community. *Participants*. Twenty-seven older female adults with sleep disturbance according to the Pittsburgh Sleep Quality Index (PSQI) >5 for at least 3 months were recruited. Participants were screened by both the Hospital Anxiety and Depression Scale (HADS) and the Mini-Mental State Examination (MMSE), as well as polysomnography prior to randomization. *Interventions*. All eligible participants were randomly allocated into the experimental or control group. Both groups were taped with magnetic press pellet on auricular points for 3 weeks. The experimental group was treated by applying pressure on the magnetic press pellets 3 times per day while no stimulation was applied on the control group. *Measurements and Results*. Both groups were measured by PSG and PSQI at the beginning of the study and 3 weeks after the study. Both groups showed improvements on PSQI scores compared to the baseline. One-way analysis of covariance adjusted for baseline scores showed that significant improvements of PSG-derived sleep parameters, such as sleep efficiency, were found in the experimental group. However, no significant differences between groups were observed in the proportion of sleep stages with the exception of Stage 2. *Conclusions*. Auricular therapy using magnetic pellets and stimulation by pressing was more effective in improving the sleep quality compared to auricular therapy without any stimulation.

## 1. Introduction

 Sleep is a periodic state of rest for the body and mind. In humans, normal sleep has five stages that cyclically repeat themselves during an episode of sleep. The first four stages comprise nonrapid eye movement (NREM) sleep and the last stage constitutes rapid eye movement (REM) sleep. Stage 1 is characterized by drowsiness, Stage 2 by light sleep, and Stages 3 and 4 by deep sleep or slow wave sleep (SWS). The NREM sleep does not occur with rapid eye movement and is associated with tissue repair. During REM sleep there are rapid periodic twitching movements of the eye muscles and accelerated respiration and heart rate, in which dreams occur and memory is thought to be organized. Hence sleep is a vital function to restore energy and maintain homeostasis of our body. However, complaints of sleep difficulties are increasing in modern society. The overall prevalence of symptoms of insomnia in adults ranged from 13.5% to 40%, with higher percentages occurring in the elderly [1–6]. The National Institute on Aging held a survey regarding sleep quality in the elderly population and found that up to 43% of respondents reported difficulty in falling asleep or maintaining sleep [7]. 

The older adults usually complain more about the sleep dissatisfaction than that in the general population [8]. Changes in sleep patterns with advancing age characterized by decreased total nocturnal sleep time and frequent nocturnal awakening often result in the older adults experiencing a lighter and more interrupted sleep [9]. Their total sleep time and sleep efficiency also decline with age [10]. Furthermore, polysomnographic studies have shown that sleep architecture which depicts the structure and pattern of sleep alters with age [9]. Compared to young adults, polysomnography-(PSG-) derived sleep architecture in older adults presents with increased NREM Stage 1 sleep and reduced amounts or absence of NREM Stage 3 and 4 sleep, indicating less deep sleep (or slow wave sleep) but more light sleep in those subjects [11, 12]. The proportion of REM sleep stays the same but the latency of REM sleep may shorten, resulting in advanced sleep phase and increase in early morning awakening [13]. Consequently, sleep disturbance is highly associated with fatigue, daytime sleepiness, psychological distress, and physical discomfort and, thereby impairing daytime functioning, quality of life as well as physical and mental health [1, 2, 14, 15].

Currently, hypnotic medication is the most popular treatment for insomnia or sleep disturbance, but some people, especially elder people, are susceptible to their adverse effects, such as daytime drowsiness, falls, drug dependence, and rebound insomnia owing to the drug's withdrawal [16]. Therefore, it is essential to explore a safe way or an alternative approach to improve the sleep quality. Evidence has shown that auricular therapies hold great potential on having positive effects on the improvement of insomnia [17–20]. Auricular therapy combines both channel theory and knowledge of nerve distributions in the auriculae to effect the autonomic nervous system and, thereby, promoting sleep quality [21, 22]. However, the paucity of rigorously designed trials was confined to reveal the real effect of auricular therapy for the treatment of insomnia [17–20]. Furthermore, most appraisals of efficacy usually use subjective experience stated as outcome measurements [23–26]. The results may fail to accurately represent the effectiveness of auricular therapy due to lack of robust objective tool measurements. Only a few studies which met the standards of clinical trial used actigraphy in the appraisal of the effectiveness of auricular therapy, but there is still lack of polysomnographic measurement, which is viewed as a golden standard for sleep assessment [27]. Also, magnetic press pellets were only placed on auricular acupoints and no stimulation was given. Nonetheless, based on the experience of traditional Chinese medicine practitioners, the magnetic press pellets placed on the acupoints must be stimulated to exert its effect. The main purpose of this study is to further examine the stimulation effect of auricular press pellet therapy on older adult women with sleep disturbance by using the polysomnography (PSG) measurements. 

## 2. Methods

### 2.1. Design

This was a randomized, single-blind, and experimental-controlled parallel-group study design to compare the effects of auricular press pellet therapy with or without stimulation. Major assessments were conducted at baseline and 3 weeks after intervention. This study was approved by the Institutional Review Board of China Medical University Hospital (DMR97-IRB-107).

### 2.2. Participants

Potential participants over 50 years of age complaining of sleep disturbance were recruited from the local community through advertisements. The inclusion criteria for selecting eligible participants were (1) 50 years of age and above; (2) complaining of sleep disturbance (difficulty in initiating sleep over 30 minutes, maintaining sleep, or awakening prematurely) 3 or more nights per week for at least 3 months; (3) having a global Pittsburgh Sleep Quality Index (PSQI) score greater than 5. In order to avoid the masking effect of hypnotics on sleep, all participants were asked not to take or to be willing to discontinue their use of any hypnotics for at least 1 week prior to the beginning of the study and during the study period.

Potential participants were excluded if they had the following factors involved: (1) sleep disturbance was relevant to obvious environmental factors; (2) a history of alcohol or substance use; (3) their sleep difficulties resulted from medical conditions, including an acute illness, unstable health conditions associated with physical discomfort or pain, malignancy ≤5 years prior to study, unstable or uncontrolled cardiac disease, diabetes, hepatic or renal disease, active endocrine disorders, nocturia (>3 times/night) and; (4) serious neurological disorders, such as stroke and Parkinson, or conditions related to impaired cognitive function, such as dementia; (5) the presence of psychiatric disorders; (6) the presence of other sleep disorders, and Apnea-Hypopnea Index (AHI) >10 or periodic limb movements (PLM); (7) persons with shift work. In addition, the Hospital Anxiety and Depression Scale (HADS) and the Mini-Mental State Examination (MMSE) were also screened to confirm participants' anxiety and depression as well as the absence of impaired cognitive function. These potential participants were also excluded if they had an HADS score >11 on either anxiety or depression subscale.

**Figure 1:**
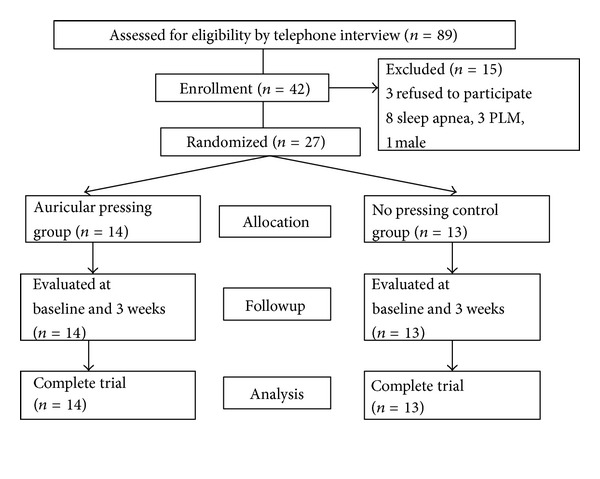
Flow of participants through the trial.

The process of selecting research participants and randomized assignments of groups were summarized in Figure 1. One hundred and eighty-eight potential participants responded and were preliminarily screened for the inclusion criteria by telephone. A total of 89 persons who met the preliminary screen were further confirmed by face-to-face interview and informed of the research process. Forty-two persons agreed to participate in this study and were scheduled for the initial PSG studies. Participants were excluded from this study if they presented with severe sleep apnea (*n* = 8), PLM (*n* = 3), were unable to adapt to the Sleep Center's environment during the first night, or refused to participate (*n* = 3). Because only one male subject remained and gender is a significant factor for sleep [28], data of only female participants were analyzed in this study. Twenty-seven female participants completed this study.

### 2.3. Procedure

After the prescreening interview, eligible participants were informed about the study and were asked to sign the consent form. They entered a sleep laboratory and underwent PSG screening to further confirm the absence of any sleep disorders, such as sleep apnea and PLM. Following the stratification by sex, qualified participants were randomly assigned to the control or experimental groups using a random digital table. Participants were not aware whether they would be placed into either the experimental group or the control group. Both groups were measured by PSG and PSQI before and after intervention.

#### 2.3.1. Auricular Therapy

Both groups were taped with a round 1.6 mm diameter magnetic press pellets similar in size to that of Semen Vaccariae on the ear acupoints. The magnetic press pellets were purchased from commercial product. Each magnetic press pellet contained an average of 100 gauss or above magnetic flux density examined by an FW Bell 4048 Gauss/Tesla Meter (made in USA). Before taping, the most sensitive auricular points were probed by forceps to further confirm the location. The participant's auricles were sterilized with 75% alcohol pads, and a magnetic press pellet was then taped to the reactive point. The magnetic press pellets and adhesive tape were replaced every 3-4 day to avoid the possibility of local irritation or ulceration on the auricular point. Both ears were taped alternately. 

#### 2.3.2. Control Group

Participants assigned to the control group were treated with no additional force applied to the magnetic pellets. 

#### 2.3.3. Experimental Group

 Participants assigned to the experimental group were given an audiotape and instructions guiding them on how to stimulate the acupoints. They were asked to apply pressure on the magnetic press pellets 3 times a day (morning, afternoon, and 30 minutes before bedtime). Each point was pressed 7 seconds and relaxed 1 second for 100 times with the exception of the point Shenmen that was pressed 120 times. Each round lasted for about 12 to 15 minutes. The pressure was exerted to the extent of obvious and tolerable feeling of pain, thereby having a swelling pain and warmth feeling on the acupoints. The trial was conducted over a 3-week period. 

#### 2.3.4. Selection of Auricular Acupoints

**Figure 2:**
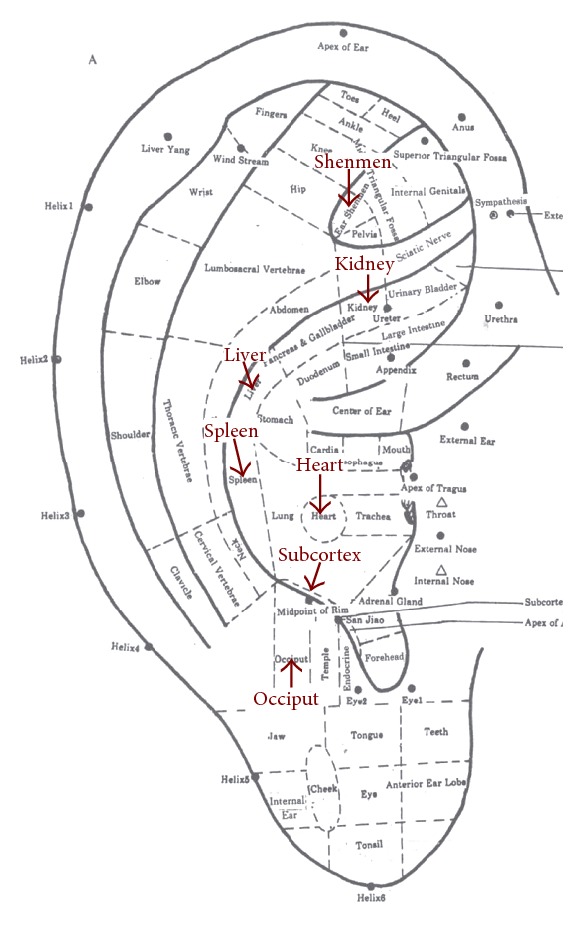
The location of selected auricular acupoints—frontal surface.

 According to Traditional Chinese Medicine (TCM), insomnia is mainly due to a dysfunction between the viscera bowels, imbalance between Yin and Yang, and hampered circulation of Qi and blood in the channels and network vessels [20]. Seven acupoints for sleep improvement were chosen based on the principles of Traditional Chinese Medicine (TCM) and a standardized protocol published by Suen et al. [29]. They are Shenmen, Heart, Kidney, Spleen, Liver, Subcortex, and Occiput. The “Heart” point can quiet the mind. The “Kidney” point supplements essence. The “Spleen” point can dispel dampness and improve digestive function. The “Liver” point can regulate the flow of Qi, especially when insomnia is caused by stagnation of liver Qi. The points “Shenmen” and “Occiput” can calm the mind. The “Subcortex” point can harmonize the excitation and inhibition of the cortex [21]. The precise auricular points are located in Figure 2 and follow the International Standard of Auricular Points [21]. 

### 2.4. Measurements

#### 2.4.1. Subjective Sleep Measures: Pittsburgh Sleep Quality Index

The Pittsburgh Sleep Quality Index [30] was used to evaluate self-rating sleep disturbance and quality at the beginning and the end of the study. Nineteen individual items composed the 7 components, including subjective sleep quality, sleep latency, sleep duration, sleep efficiency, sleep disturbances, use of sleeping medication, and daytime dysfunction, to evaluate participants' sleep over a 1-month time interval. According to the Likert scale, each component was scored 0–3 points, and the sum of these component scores generated a global PSQI score ranging from 0 to 21. The overall Cronbach alpha of the Chinese version of the Pittsburgh Sleep Quality Index was 0.82 [31]. The higher scores indicated poor sleep quality. A score of 5 or greater was used to differentiate between poor and good sleep. Participants had to score >5 to be eligible for this study.

#### 2.4.2. Objective Sleep Measures: Polysomnography (PSG)

 Polysomnography is considered a gold standard in assessing sleep status. It can record electroencephalogram (EEG), electrooculogram (EOG), electromyogram (EMG), and electrocardiography (ECG) as well as breathing patterns at the same time. Data was obtained using the Alice IV sleep data acquisition system (Respironics, Murrysville, PA, USA). Two consecutive overnight PSG were performed in the sleep center at the beginning and the end of the 3-week period of auricular therapy. PSG results obtained on the first night during the before and after study were not included in the analysis in order to avoid first night effect. Sleep stage scoring of the entire PSG recordings were visually analyzed by a well-trained sleep technician according to the Rechtschaffen and Kales standard. Sleep parameters included sleep latency (SL, lights off to the first epoch of Stage 1 sleep), total sleep time (TST, the sum of sleep Stages 1–4 and REM), sleep period time (SPT, time from the beginning of NREM Stage 1 until final awakening), sleep efficiency (SE, ratio of TST/TIB), waking after sleep onset (WASO), and arousal index. Sleep architecture was also obtained, including Stage 1 to 4 of nonrapid eye movement (NREM) sleep, slow wave sleep (SWS, indicating the deep sleep), and rapid eye movement (REM) sleep [32].

#### 2.4.3. Sleep Apnea and Periodic Limb Movements (PLM)

Screening for evidence of sleep apnea and periodic limb movements (PLM) was done during the first night in the sleep center. The evaluation criteria were based on the Report of the American Academy of Sleep Medicine Task Force [33]. The Apnea-Hypopnea Index (AHI) <10 was used as the screening criterion for this study. 

### 2.5. Statistical Analysis

Descriptive statistics were used to summarize the participants' demographic characteristics. Demographic differences between the experimental and control groups were examined using either *t*-test or *χ* ^2^ test. Mean values of PSG sleep parameters (TST, SL, SE, WASO, and sleep stage percents and minutes) and perceived sleep quality (PSQI) were calculated. A one-way analysis of covariance (ANCOVA) with baseline measure as covariate was used to compare changes in PSG and perceived sleep parameters between the experimental and control groups after intervention. Each analysis was performed at the significance level of *P* < 0.05. All statistical data was carried out by using SPSS 17.0 software for windows.

## 3. Results

### 3.1. Demographic Data

The demographic information of participants was summarized in Table 1. Their age ranged from 50 to 68 years, with an average of 57 and 56.8 years, respectively, for the experimental group and control group. Average of BMIs of both groups fell within the normal range. There was no difference between two groups in various screening indicators of all participants, such as the degree of cognitive and the level of anxiety and depression. Most participants were married and lived with their spouses or children. In both groups approximately 30% to 40% of the participants were retired or worked as a volunteer with an education level of middle school or below. The duration of insomnia was 92 months in the experimental group and up to 105 months in the control group without significant differences between groups. There were no significant differences in the variance of demographic variables between groups, indicating homogeneous and comparable groups.

### 3.2. Changes in Subjective Sleep Quality (PSQI) between Pre- and Posttests

Table 2 illustrated the results of the comparative analysis of both groups' PSQI scores using analysis of covariance (ANCOVA). There are no differences between the groups in the global PSQI score as well as the 7 components observed at baseline. After intervention, the mean global score of PSQI reached significant difference (*F*_(1,23)_ = 9.10, *P* < 0.01) between groups, indicating that sleep quality of the experimental group improved more than that of the control group. Significant differences (*P* < 0.05) also existed between two groups within the detailed components of PSQI in the posttest, including sleep quality, sleep duration, sleep efficiency, sleep disturbances, and daytime dysfunction. 

### 3.3. Changes in Polysomnography between Pre- and Posttests

**Figure 3:**
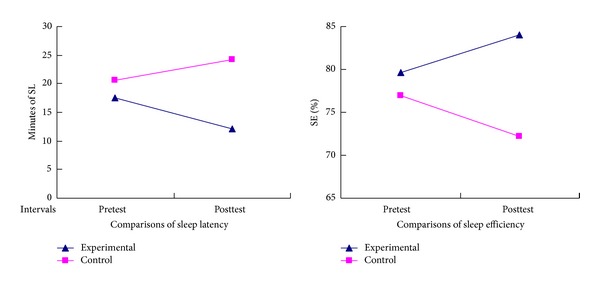
The changes of sleep latency and sleep efficiency detected by PSG between the experimental group and the control group.

 Sleep parameters measured by PSG were summarized in Table 3. The changes of sleep latency and sleep efficiency detected by PSG between the experimental group and the control group were also illustrated in Figure 3. The results of the comparative analysis in both groups' sleep parameters were analyzed by analysis of covariance (ANCOVA). Prior to auricular therapy, older adults in both groups had sleep efficiency less than 80%, more than 15 minutes of sleep latency, and arousal index up to 15 times per hour, indicating poor sleep quality in both groups. There were no significant differences in all PSG sleep parameters between groups at the pretest. After intervention, sleep efficiency was increased sleep latency, WASO, and arousal index were decreased significantly (*P* < 0.05) in the experimental group.

Comparing the sleep architecture, no significant differences existed in the parameters of each sleep stages between the two groups at the pretest. After intervention, minutes and percentages of Stage 1 sleep were decreased in the experimental group while increased in the control group. There was no statistical difference in the proportion and duration of other sleep stages between groups with the exception of Stage 2. 

## 4. Discussion

 Previous studies have shown that auricular therapy with magnetic press pellets improved subjective sleep quality in patients of all ages [25, 34, 35]. Our study further showed that auricular therapy using magnetic press pellets effectively improved the sleep quality evaluated by both subjective sleep and polysomnography in older community dwelling women with sleep disturbance. Therefore, auricular therapy can be an effective way to improve sleep quality in women with sleep disturbance.

Compared to auricular therapy of using magnetic press pellets without stimulation by pressing, total sleep time and sleep efficiency are increased as well as sleep latency, wake minutes, and Stage 1 sleep are decreased in those of using magnetic press pellets with stimulation. The effects of the auricular magnetic press pellets with stimulation on both subjective and objective sleep qualities are better than that without auricular stimulation. Previous studies have shown that the effect of using magnetic press pellets without stimulation in auricular therapy is better than that of using Junci Medulla or Semen Vaccariae [27]. It suggested that the effectiveness of auricular therapy on sleep improvement is due to the magnetic effect. However, our study further showed that even magnetic press pellets used in auricular therapy are more efficacious when the magnetic press pellets are stimulated against the acupoints. Stimulation is also essential in auricular therapy. Magnetic press pellets with stimulation evoke better sleep improvement.

 Most of the previous studies used the subjective feeling of patients to appraise the effectiveness of improvement in sleeping. In our study we emphasized using the PSG instrument in order to objectively evaluate the outcome and effect of auricular therapy, since PSG is viewed as the gold standard to assess sleep and can obtain more comprehensive and objective information. Interestingly, it was found that the level of improvement of sleep quality appraised by PSG was far less than that by subjective feeling. Therefore, the limitation of Hawthorne effect and social desirability bias effect cannot be ignored in subjective appraisal. By using both subjective and objective sleep, assessment is necessary in evaluating the real effect of sleep interventions.

The findings of this study are consistent with the fact that the elderly easily experienced lighter and more fragmented sleep [9, 10]. The results showed that older women with sleep disturbance had difficulties in falling asleep and needed greater than 15 minutes to enter Stage  1 before the auricular therapy. The patients' sleep pattern fluctuated back and forth repeatedly between Stage 1 and Stage 2. This was also accompanied by significantly increased arousal event of up to 15–17 times per hour in the entire night. This led them to remain in a state of light sleep which augments the disruption of sleep. As a result, older women with sleep disturbance could not experience better quality of sleep.

After auricular stimulation, sleep latency became less than 15 minutes in the experimental group, which was obviously shorter than that of the nonstimulation control group. In the findings of the PSG appraisal, the improvement of sleep efficiency of the experimental group increased to 84.04%, but sleep efficiency of the control group did not improve distinctly. Besides, in the aspect of sleep disturbance, indicator of wakefulness (e.g., WASO, arousal index) of the experimental group showed a statistically significant improvement compared to the control group. This showed that with progression of insomnia, intervention of the experimental group can better improve sleep quality. Fascinatingly, in the aspect of sleep architecture, though the two groups show no observable difference in stages of deep sleep (Stages 3 and 4), Stage 1 sleep of the experimental group decreased and transferred to Stage 2 sleep and REM sleep. Auricular stimulation may urge sleep to develop from shallow sleep to deeper intermediate sleep while the number of arousal event significantly decreased, thereby improving sleep quality.

Though the effectiveness of how auricular stimulation can improve sleep quality was not as much as we expected, this procedure is relatively simple and easy to perform. Therefore, it can be considered to be a complementary alternative therapy. The probable mechanism for the improvement of sleep by stimulating the auricle in this study could be explained based on several physiological functions. There are many nerve innervations in the auricle, such as the great auricular nerve, and lesser occipital nerve from the cervical plexus of the spinal ganglia; branches of the ear temporal nerve, facial, glossopharyngeal nerve and vagus nerve from the cranial nerves; and sympathetic nerves which follow the external carotid artery [21]. Therefore, through the regulatory function of the central nervous system, the stimulation of the auricular points plays a regulatory role in the treatment of insomnia. However, stimulation of the auricle is a simple complementary alternative therapy, yet the compliance of the participants towards self-stimulation should be considered. Otherwise, the extent of stimulation could affect the effect of sleep improvement. Although this study used randomized controled trial and a single-blind design, there were still some limitations in this study. One of the limitations is the small number of participants studied; therefore future studies will require a larger number of participants to substantiate our findings. Another limitation is that the auricular stimulation and outcome measurement were performed by the same investigator, and, thus, the interaction between the investigator and participants may cause subjective measurement bias. The social desirability and Hawthorne effect were difficult to be avoided. Further double-blinded studies are required to determine whether subjective effects existed in the current single-blind study. Moreover, a long-term follow-up study with an increased range of ages and male participants will be necessary to ascertain the effectiveness of auricular stimulation.

## Figures and Tables

**Table 1:** Demographic data of participants in the experimental and control group.

Variables	Experiment (*n* = 14)	Control (*n* = 13)
	Mean ± SD	Mean ± SD
Age, years	57.0 ± 4.6	56.8 ± 4.9
Insomnia duration, month	92.4 ± 84.39	105.5 ± 102.83
BMI^#^, %	22.59 ± 2.74	22.56 ± 2.98
MMSE^#^	29.2 ± 0.9	28.9 ± 1.3
HADS^#^_anxiety	3.6 ± 3.3	3.4 ± 2.5
HADS^#^_depression	2.9 ± 2.7	2.9 ± 2.4

	*n*/(%)	*n*/(%)

Retired	4 (28.6%)	5 (38.5%)
Education level		
Middle school or below	4 (28.6%)	5 (38.5%)
High school	6 (42.8%)	6 (46.2%)
College or above	4 (28.6%)	2 (15.4%)
Marriage: married	11 (78.6%)	10 (76.9%)
Religion		
None or other	2 (14.2%)	2 (15.4%)
Buddhist	6 (42.9%)	5 (38.5%)
Folk religion	6 (42.9%)	6 (46.2%)
Living status		
With spouse	4 (28.6%)	1 (7.7%)
With children	3 (21.4%)	7 (53.8%)
With spouse and children	5 (35.7%)	4 (30.8%)
Other	2 (14.3%)	1 (7.7%)
Other family members suffering from insomnia	6 (42.9%)	7 (53.8%)

^#^BMI: body mass index; MMSE: Mini-Mental State Examination; HADS: Hospital Anxiety and Depression Scale.

**Table 2:** Comparisons of seven components and global score of PSQI between the experimental group and the control group by analysis of covariance (ANCOVA).

Items of PSQI	Pretest		Posttest		ANCOVA	
	Experiment (*n* = 14)	Control (*n* = 13)	Experiment (*n* = 14)	Control (*n* = 13)	*F *	*P* value
	Mean ± SD	Mean ± SD	Mean ± SD	Mean ± SD		
C1. Sleep quality	2.47 ± 0.52	2.46 ± 0.52	0.80 ± 0.56	1.31 ± 0.75	4.73	0.04*
C2. Sleep latency	2.87 ± 0.52	2.69 ± 0.63	1.33 ± 0.82	1.77 ± 0.73	2.48	0.13
C3. Sleep duration	2.53 ± 0.64	2.54 ± 0.66	1.07 ± 0.70	1.92 ± 0.95	5.17	0.03*
C4. Sleep efficiency	2.33 ± 0.82	2.62 ± 0.77	0.80 ± 1.15	1.85 ± 1.14	5.41	0.02*
C5. Sleep disturbances	1.00 ± 0.38	1.08 ± 0.28	0.73 ± 0.46	1.15 ± 0.38	7.06	0.01*
C6. Sleeping medication	1.07 ± 1.28	0.77 ± 1.24	0.00 ± 0.00	0.15 ± 0.38	0.84	0.37
C7. Daytime dysfunction	1.20 ± 1.08	1.54 ± 0.97	0.33 ± 0.62	1.08 ± 0.95	4.47	0.04*
PSQI global score	13.40 ± 2.90	13.69 ± 1.97	5.07 ± 2.63	9.23 ± 3.63	9.10	0.00**

*P < 0.05; **P < 0.01.

**Table 3:** Comparisons of the sleep parameters detected by PSG between the experimental group and the control group by analysis of covariance (ANCOVA).

Sleep parameters	Pretest		Posttest		ANCOVA	
	Experiment (*n* = 14)	Control (*n* = 13)	Experiment (*n* = 14)	Control (*n* = 13)	*F *	*P* value
	Mean ± SD	Mean ± SD	Mean ± SD	Mean ± SD		
Sleep latency (to Stage 1) (min)	17.57 ± 11.27	20.58 ± 13.13	12.04 ± 6.74	24.27 ± 15.48	6.78	0.02*
Total sleep time (min)	356.29 ± 51.61	335.65 ± 54.97	362.14 ± 31.46	314.31 ± 66.96	5.87	0.02*
Sleep period time (min)	423.75 ± 45.67	416.00 ± 49.21	409.18 ± 35.34	401.62 ± 41.89	0.18	0.67
SE^#^ (TST/TIB) (%)	79.58 ± 7.54	76.99 ± 12.62	84.04 ± 4.62	72.15 ± 12.78	10.85	0.00**
Sleep stages (min in sleep period time)						
Wake (WASO^#^)	61.75 ± 38.72	80.35 ± 48.76	47.04 ± 24.07	87.31 ± 50.42	6.90	0.02*
Stage 1	54.82 ± 42.87	50.85 ± 29.74	42.93 ± 15.60	60.62 ± 50.58	2.22	0.15
Stage 2	208.43 ± 56.22	187.23 ± 41.12	208.46 ± 47.28	158.15 ± 54.67	6.29	0.02*
Stage 3	10.21 ± 15.31	14.62 ± 20.65	11.64 ± 15.45	15.92 ± 21.01	0.17	0.69
Stage 4	0.93 ± 3.06	4.08 ± 12.51	0.64 ± 1.99	2.81 ± 8.50	0.48	0.50
REM	87.61 ± 27.93	78.88 ± 23.55	98.46 ± 34.01	76.81 ± 26.24	2.08	0.16
NREM	274.39 ± 30.12	256.77 ± 42.75	263.68 ± 42.71	237.50 ± 52.26	0.56	0.46
SWS	11.14 ± 18.11	18.69 ± 31.40	12.29 ± 16.64	18.73 ± 27.83	0.02	0.89
Sleep stages (**%** in sleep period time)						
Wake (WASO^#^)	14.36 ± 8.03	19.17 ± 11.28	11.34 ± 5.36	21.96 ± 13.20	6.74	0.02*
Stage 1	13.03 ± 9.89	11.96 ± 6.34	10.43 ± 3.52	15.15 ± 13.16	1.98	0.17
Stage 2	49.50 ± 13.27	45.80 ± 11.54	50.90 ± 10.27	39.18 ± 12.24	4.75	0.04*
Stage 3	2.35 ± 3.52	3.03 ± 4.39	2.86 ± 3.73	3.90 ± 5.00	0.03	0.87
Stage 4	0.21 ± 0.72	0.79 ± 2.34	0.14 ± 0.45	0.68 ± 2.03	0.03	0.87
REM	21.21 ± 4.79	19.04 ± 5.48	24.31 ± 8.61	19.12 ± 6.35	2.73	0.11
Arousal index	14.57 ± 5.01	15.27 ± 2.65	12.99 ± 5.00	16.78 ± 3.97	4.81	0.04*

^#^SE: sleep efficiency; WASO: wake after sleep onset.

*P < 0.05; **P < 0.01.

## References

[1] Kao CC, Huang CJ, Wang MY, Tsai PS (2008). Insomnia: prevalence and its impact on excessive daytime sleepiness and psychological well-being in the adult Taiwanese population. *Quality of Life Research*.

[2] Chiu HF, Leung T, Lam LC (1999). Sleep problems in Chinese elderly in Hong Kong. *Sleep*.

[3] Kim K, Uchiyama M, Okawa M, Liu X, Ogihara R (2000). An epidemiological study of insomnia among the Japanese general population. *Sleep*.

[4] Montgomery P, Lilly J (2007). Insomnia in the elderly. *Clinical Evidence*.

[5] Morphy H, Dunn KM, Lewis M, Boardman HF, Croft PR (2007). Epidemiology of insomnia: a longitudinal study in a UK population. *Sleep*.

[6] Sivertsen B, Krokstad S, Øverland S, Mykletun A (2009). The epidemiology of insomnia: associations with physical and mental health. The HUNT-2 study. *Journal of Psychosomatic Research*.

[7] Foley DJ, Monjan AA, Brown SL, Simonsick EM, Wallace RB, Blazer DG (1995). Sleep complaints among elderly persons: an epidemiologic study of three communities. *Sleep*.

[8] Pallesen S, Nordhus IH, Nielsen GH (2001). Prevalence of insomnia in the adult Norwegian population. *Sleep*.

[9] Feinsilver SH (2003). Sleep in the elderly. What is normal?. *Clinics in Geriatric Medicine*.

[10] Hoffman S (2003). Sleep in the older adult: implications for nurses (CE). *Geriatric Nursing*.

[11] Floyd JA, Medler SM, Ager JW, Janisse JJ (2000). Age-related changes in initiation and maintenance of sleep: a meta-analysis. *Research in Nursing and Health*.

[12] van Someren V, Burmester M, Alusi G, Lane R (2000). Are sleep studies worth doing?. *Archives of Disease in Childhood*.

[13] Ancoli-Israel S (2000). Insomnia in the elderly: a review for the primary care practitioner. *Sleep*.

[14] Hidalgo JL, Gras CB, Garcia YD, Lapeira JT, del Campo del Campo JM, Verdejo MA (2007). Functional status in the elderly with insomnia. *Quality of Life Research*.

[15] Morin CM, LeBlanc M, Daley M, Gregoire JP, Mérette C (2006). Epidemiology of insomnia: prevalence, self-help treatments, consultations, and determinants of help-seeking behaviors. *Sleep Medicine*.

[16] (2008). Sleep complaints: whenever possible, avoid the use of sleeping pills. *Prescrire International*.

[17] Hai YC, Shi Y, Chi SN, Sai MC, Yung KKL, Qing LZ (2007). Auricular acupuncture treatment for insomnia: a systematic review. *Journal of Alternative and Complementary Medicine*.

[18] Lee MS, Shin B-C, Suen LKP, Park T-Y, Ernst E (2008). Auricular acupuncture for insomnia: a systematic review. *International Journal of Clinical Practice*.

[19] Huang W, Kutner N, Bliwise DL (2009). A systematic review of the effects of acupuncture in treating insomnia. *Sleep Medicine Reviews*.

[20] Shen P (2004). Two hundred cases of insomnia treated by otopoint pressure plus acupuncture. *Journal of Traditional Chinese Medicine*.

[21] Feng CX, Bai XH, Du Y (1994). *Chinese Auricular Therapy (Chinese-English Version)*.

[22] Gori L, Firenzuoli F (2007). Ear acupuncture in European traditional medicine. *Evidence-Based Complementary and Alternative Medicine*.

[23] Gao Y (1995). Insomnia treated by auricular acupressure in 128 cases. *Shanghai Journal of Acupuncture and Moxibustion*.

[24] Jin RF (2003). Stimulating auricular acupoint by Semen Vaccariae to treat internal diseases complicated with insomnia in 64 cases. *Zhejiang Journal of Traditional Chinese Medicine*.

[25] Wang ST (2002). An observation on efficacy of auricular acupressure on insomnia using magnetic press pellets. *Anhui Clinical Journal of Traditional Chinese Medicine*.

[26] Zhang J, Li J, Li YS, Pan LL, Zhang M (2000). A controlled study of treating insomnia by auricular acupressure and diazepam. *Yunan Journal of Traditional Chinese Medicine and Materia Medica*.

[27] Suen LKP, Wong TKS, Leung AWN (2002). Effectiveness of auricular therapy on sleep promotion in the elderly. *American Journal of Chinese Medicine*.

[28] Zhang B, Wing Y-K (2006). Sex differences in insomnia: a meta-analysis. *Sleep*.

[29] Suen LKP, Wong TKS, Leung AWN (2002). Auricular therapy using magnetic pearls on sleep: a standardized protocol for the elderly with insomnia. *Clinical Acupuncture and Oriental Medicine*.

[30] Buysse DJ, Reynolds CF, Monk TH, Berman SR, Kupfer DJ (1989). The Pittsburgh sleep quality index: a new instrument for psychiatric practice and research. *Psychiatry Research*.

[31] Tsai PS, Wang SY, Wang MY (2005). Psychometric evaluation of the Chinese version of the Pittsburgh sleep quality index (CPSQI) in primary insomnia and control subjects. *Quality of Life Research*.

[32] Landis CA (2002). Sleep and methods of assessment. *Nursing Clinics of North America*.

[33] Medicine AAoS (2007). *The AASM Manual for the Scoring of Sleep and Associated Events*.

[34] Lu W (2000). Comparsion between megnetic pearls and Semen Vaccariae on auricular acupressing treating insomnia in 108 cases. *Chinese Acupuncture & Moxibustion*.

[35] Qian YJ (2005). Treating insomnia with auricular acupressing using megnatic perals. *Shanghai Journal of Acupuncture and Moxibustion*.

